# Acetylation Regulates Thioredoxin Reductase Oligomerization and Activity

**DOI:** 10.1089/ars.2017.7082

**Published:** 2018-08-01

**Authors:** David E. Wright, Zaid Altaany, Yumin Bi, Zaccary Alperstein, Patrick O'Donoghue

**Affiliations:** ^1^ Department of Biochemistry, The University of Western Ontario, London, Canada.; ^2^ Department of Basic Sciences, Faculty of Medicine, Yarmouk University, Irbid, Jordan.; ^3^ Department of Chemistry, The University of Western Ontario, London, Canada.

**Keywords:** orthogonal translation, post-translational modification, redox, selenoprotein, tRNA^Sec^, tRNA^Pyl^

## Abstract

***Aims:*** Thioredoxin reductase 1 (TrxR1) is a cancer target and essential selenoprotein that defends the cell against reactive oxygen species and regulates cellular signaling and redox pathways. Previous cell-based studies correlated TrxR1 acetylation with modulated cellular reduction activity, yet the function of specific acetylation sites on TrxR1 remains unknown.

***Innovation:*** We produced site-specifically acetylated TrxR1 variants that also contain selenocysteine (Sec). We demonstrated efficient high-fidelity protein synthesis with 22 different amino acids by simultaneous UAG codon reassignment to *N_ɛ_*-acetyl-lysine and UGA codon recoding to Sec.

***Results:*** We characterized TrxR1 variants acetylated at physiologically relevant sites and found that single acetylation sites increased TrxR1 activity, enhancing the apparent catalytic rate up to 2.7-fold. The activity increase in acetylated TrxR1 (acTrxR1) is reversible and is reduced following deacetylation with histone deacetylase.

***Conclusion:*** Here we present a novel mechanism through which acetylation increases TrxR1 activity by destabilizing low-activity TrxR1 multimers, increasing the population of active dimeric TrxR1. *Antioxid. Redox Signal.* 29, 377–388.

## Introduction

Human cells naturally defend against reactive oxygen species (ROS) using a network of redox enzymes, including the selenoprotein thioredoxin reductase 1 (TrxR1) (38). Selenoproteins contain the 21st genetically encoded amino acid, selenocysteine (Sec). In cells, the Sec550 residue in TrxR1 is required for most of its activity (42) in reducing oxidized compounds, for example, ubiquinone (53), and oxidatively damaged cellular proteins *via* a redox-coupled reaction with thioredoxin (Trx). TrxR1 catalyzes the transfer of electrons from β-nicotinamide adenine dinucleotide 2′-phosphate (NADPH) to Trx1. The reduced Trx1 in turn resolves oxidized species and reduces cellular proteins. The resulting oxidized Trx1 is then recycled by the TrxR1 enzyme. In addition to ROS defense, the Trx system is involved in regulating gene expression, embryonic development, cell proliferation, and apoptosis (27).

Innovation Proteins are normally synthesized in cells with 20 amino acids, but most are post-translationally modified at many sites with unknown consequences to protein function. We used two mutually orthogonal translation systems to incorporate 22 genetically encoded amino acids into a single protein with efficiency and fidelity identical to normal protein synthesis. In doing so, we produced a purified human enzyme in a native and more fully active form, and importantly, we uncovered acetylation as a novel mechanism of enhancing thioredoxin reductase 1 activity that has broad implications for cellular signaling and disease pathways related to deregulation of the cellular redox status.

Overactive TrxR1 is associated with chemotherapeutic resistance, and TrxR1 activity is co-opted by cancer cells to defend against ROS generated by therapeutic compounds (35). TrxR1 activity levels are diagnostic for early detection of lung (39) and breast cancers (8), and TrxR1 has emerged as a target to combat drug-resistant lung cancers, for example, ethaselen (49). In mouse models of age-related macular degeneration and glaucoma, overexpression of amyloid β was associated with increased TrxR1 activity despite unperturbed TrxR1 protein abundance, implicating post-translational modifications in regulating TrxR1 activity (25). Differential total TrxR activity and changes in TrxR1 acetylation status were also associated with a cardiomyopathy phenotype in transgenic mice (2) and in retinal tissue from diabetic rat models and human postmortem patient samples (26). TrxR1 is acetylated on at least three specific sites on the protein surface that have been experimentally identified in humans and mice (7, 21, 50).

**FIG. 1.**
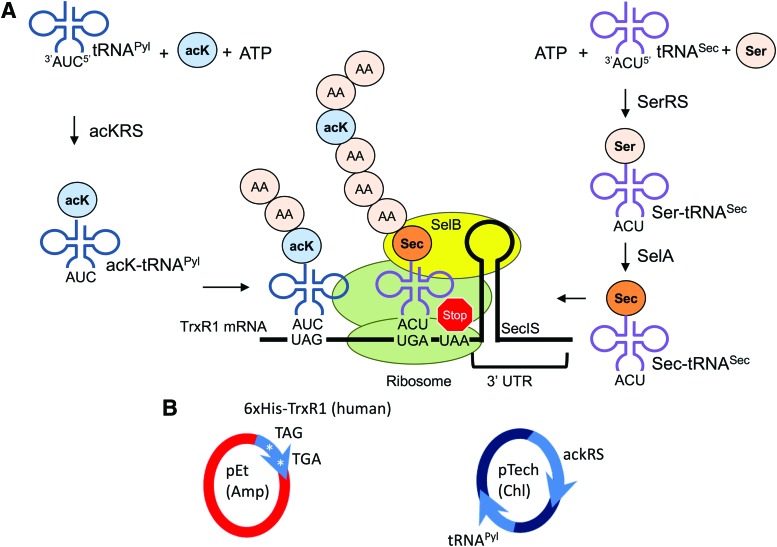
**Schematic for genetically encoding 22 amino acids. (A)** Genetically encoding acK and Sec: acKRS ligates acK to tRNA^Pyl^, which reassigns all UAG stop codons to acK. This allows insertion of acK at a specific UAG codon in TrxR1. For Sec insertion, tRNA^Sec^ is aminoacylated with serine (Ser) by seryl-tRNA synthetase (SerRS), followed by conversion of Ser-tRNA to Sec-tRNA by selenocysteine synthase (SelA). The elongation factor SelB binds Sec-tRNA^Sec^ as well as the SecIS, which we designed into the 3′ UTR of the TrxR1 mRNA. The SecIS localizes Sec-tRNA^Sec^ at the UGA codon, allowing for Sec insertion at a single UGA stop codon. **(B)** The two-plasmid system required for production of acetylated TrxR1: the pTech vector expresses AcKRS and tRNA^Pyl^, required for reassignment of UAG codons to acK. The pET vector expresses TrxR1 with a TAG at the desired acetylation sites (141, 200, or 307), as well as SecIS in the 3′ UTR (see the “Materials and Methods” section). In this system, UAG genetically encodes acK, while SecIS following downstream of the UGA codon is required for Sec insertion. The Sec synthesis and insertion system is endogenous to *Escherichia coli*, encoded in the *E. coli* genome (SelA, SelB, SelC, SelD, SerRS). 3′ UTR, 3′ untranslated region; acK, *N_ɛ_*-acetyl-lysine; acKRS, mutant pyrrolysyl-tRNA synthetase (*N_ɛ_*-acetyl-lysine-tRNA synthetase); Sec, selenocysteine; SecIS, selenocysteine insertion sequence; TrxR1, thioredoxin reductase 1. To see this illustration in color, the reader is referred to the web version of this article at www.liebertpub.com/ars

Due to the emerging association of TrxR1 acetylation, cellular redox status, and disease, we investigated how acetylation signaling modulates TrxR1 activity. Because the acetyl transferase(s) that acetylate TrxR1 in the cell are unknown, it was not possible to produce site-specifically acetylated TrxR1 variants using an upstream enzyme or from expression in human cell culture. We overcame this barrier by using two genetic code expansion systems (Fig. 1) that enabled cotranslational incorporation of both Sec and *N_ɛ_*-acetyl-lysine (acK) residues at specific sites, resulting in site-specifically acetylated and differentially active human TrxR1 selenoprotein variants.

## Results

To biochemically characterize the impact of site-specific acetylation on the reduction activity of TrxR1, we produced TrxR1 variants in *Escherichia coli* with 22 different genetically encoded amino acids, including the noncanonical amino acids (ncAAs) acK and Sec (Fig. 1). The data show, for the first time, that we produced protein with 22 different amino acids with equal efficiency and fidelity to protein production with the 20 standard amino acids (Table 1).

**Table 1.** Thioredoxin Reductase 1 and acetylated Thioredoxin Reductase 1 Protein Yields and Activity

			*DTNB assay*		*9,10-Phenanthrequinone assay*		*Insulin linked Trx1 assay*	
*TrxR1 variant*	*ΔRF1, protein yield, (mg/L)*	*BL21, protein yield, (mg/L)*	*Initial velocity (pmol TNB/s)*	*Relative activity (x-fold)*	*Initial velocity (pmol NADPH consumed/s)*	*Relative activity (x-fold)*	*Initial velocity (pmol NADPH consumed/s)*	*Relative activity (x-fold)*
WT TrxR1	0.4	2.4	0.24 ± 0.01	1.0	1.23 ± 0.03	1.0	0.22 ± 0.02	1.0
acTrxR1^K141^	0.4	0.7	0.42 ± 0.03	1.8	1.53 ± 0.05	1.2	0.41 ± 0.01	1.8
acTrxR1^K200^	0.5	2.6	0.49 ± 0.01	2.1	1.78 ± 0.14	1.4	0.42 ± 0.01	1.9
acTrxR1^K307^	0.6	0.7	0.64 ± 0.07	2.7	1.61 ± 0.08	1.3	0.43 ± 0.01	2.0
inactive TrxR1^U550Y^	n.d.	2.1	n.d.	—	—	—	n.d.	—

Protein yield was measured using the Bradford assay and noted above in milligrams of TrxR1 produced per liter of *Escherichia coli* culture from the indicated strain.

acTrxR1, acetylated thioredoxin reductase 1; DTNB, 5,5′-dithiobis-(2-nitrobenzoic acid); NADPH, β-nicotinamide adenine dinucleotide 2′-phosphate; n.d., not determined; RF1, release factor 1; Trx1, thioredoxin 1; TrxR1, thioredoxin reductase 1; WT, wild type

### *E. coli* host strain alters expression of TrxR1 with 22 amino acids

We combined two genetic code expansion systems in *E. coli* to produce site-specifically acetylated TrxR1 variants (Fig. 1). We reassigned UAG codons from stop to acK using a mutant UAG-decoding pyrrolysyl-tRNA synthetase (acKRS) and tRNA^Pyl^ pair (12, 16, 45), and we simultaneously recoded a specified UGA codon from stop to Sec with the native *E. coli* Sec insertion system (Fig. 1). We produced and purified wild-type (WT) TrxR1 with Sec at position 550, and three site-specifically acetylated variants (acTrxR1^K141^, acTrxR1^K200^, acTrxR1^K307^) (Supplementary Fig. S1; Supplementary Data are available online at www.liebertpub.com/ars). Production of these protein variants required the two-plasmid system depicted in Figure 1 and described in the Materials and Methods section.

We tested the expression of TrxR1 and acetylated TrxR1 (acTrxR1) variants in *E. coli* BL21 DE3 and in an *E. coli* strain (C321.ΔA.exp) that lacks release factor 1 (RF1) and all genome encoded UAG codons (*E. coli* ΔRF1) (24). RF1 terminates translation at UAG. *E. coli* ΔRF1 should allow for more efficient reassignment of UAG to acK, since there is less competition between acK-tRNA^Pyl^ and RF1 for binding UAG. With the acK incorporation system (Fig. 1A), we demonstrated efficient UAG read-through per cell using a GFP reporter in *E. coli* ΔRF1 (Supplementary Fig. S2). Unfortunately, and concordant with our previous work (14), the *E. coli* ΔRF1 strain produces significantly less recombinant TrxR1 (Table 1) due to phenotypic defects resulting in slow growth and markedly reduced cell densities (14, 24). Potential benefits from RF1 deletion are subsumed by the more efficient protein producing stain BL21, which produces more overall UAG translation per l *E. coli* culture.

Fascinatingly, BL21 protein production efficiency was indistinguishable (2–2.6 mg/L culture) for variants of TrxR1 containing 20 (Sec550Tyr), 21 (WT), or 22 (acTrxR1^K200^) genetically encoded amino acids (Table 1). In BL21, we observed yields for protein containing 22 genetically encoded amino acids of up to 2.6 mg/L culture, whereas the *E. coli* ΔRF1 showed a maximal protein production of 0.6 mg/L culture. BL21 produced 1.2- to 5-fold more TrxR1 or acTrxR1 compared to *E. coli* ΔRF1 (Table 1).

### Physical characterization of TrxR1 variants

**FIG. 2.**
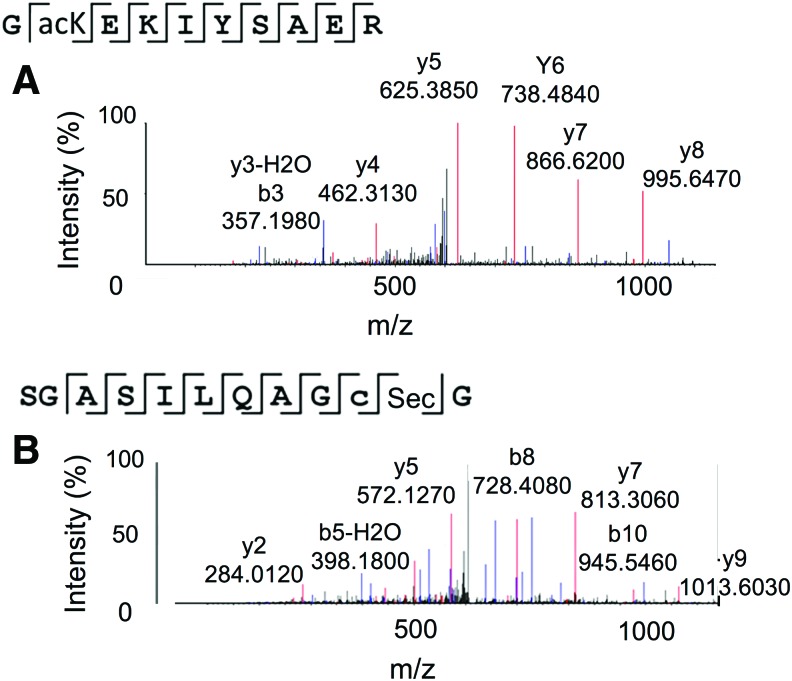
**Physical identification of acK and Sec in** **acTrxR1^K200^.** Mass spectrometry data for other TrxR1 variants are shown in Supplementary Figures S3–S6. Liquid chromatography tandem mass spectrometry of polypeptides from trypsin digested acTrxR1^K200^, demonstrating **(A)** acK and **(B)** Sec incorporation at the desired locations. acTrxR1, acetylated TrxR1. To see this illustration in color, the reader is referred to the web version of this article at www.liebertpub.com/ars

We confirmed insertion of Sec and acK into the same protein variants by multiple independent mass spectroscopic methods. Independent mass spectrometry (MS) techniques confirmed quantitative incorporation of Sec and/or acK in each TrxR1 variant (Fig. 2 and Supplementary Fig. S3–S6). Matrix-assisted laser desorption/ionization mass spectrometry (MALDI-MS) analysis of the acTrxR1 variants confirmed site-specific acetylation at each of the anticipated sites (Supplementary Fig. S3), and verified Sec incorporation in WT TrxR1 (Supplementary Fig. S4). In addition, we found no evidence of deacetylation in the MALDI data (Supplementary Fig. S3). To verify Sec incorporation and to determine the number of active molecules in each sample, we quantified the amount of selenium (Se) in the TrxR1 variants. Because Sec is required for TrxR1 activity, the amount of Se covalently bound to the protein indicates the amount of active TrxR1. Inductively coupled plasma mass spectrometry (ICP-MS) verified quantitative insertion of Sec in the TrxR1 variants (Supplementary Table S1). Liquid chromatography tandem mass spectrometry (LC-MS/MS) revealed acK incorporation (Fig. 2 and Supplementary Fig. S6) and Sec incorporation (Fig. 2 and Supplementary Fig. S5) at the programmed locations for acTrxR1^K141^ (Supplementary Figs. S5B and S6A), acTrxR1^K200^ (Fig. 2), and acTrxR1^K307^ (Supplementary Figs. S5C and S6B), as well as Sec incorporation for WT TrxR1 (Supplementary Fig. S5A). In each MS experiment, we were unable to detect deacetylation or mistranslation at the UAG- or UGA-encoded loci.

### Reversible activation of TrxR1

The enzymatic activity of TrxR1 was assessed on three substrates: 5,5′-dithiobis-(2-nitrobenzoic acid) (DTNB), 9,10-phenanthrequinone, and Trx1 in an insulin coupled assay (28). DTNB is a long-established artificial substrate for TrxR1 (28), while 9,10-phenanthrequinone and the Trx1 insulin linked assays are dependent on the presence of Sec in TrxR1 (4). DTNB and the quinone assays measure TrxR1 reduction activity directly, while the insulin linked assay also includes Trx1 and recapitulates a model TrxR1 redox pathway *in vitro*.

**FIG. 3.**
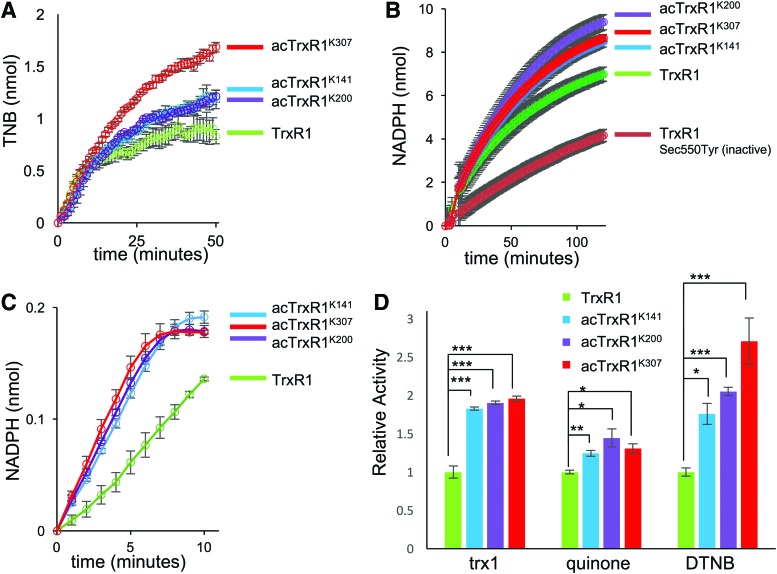
**Activity of TrxR1 and acTrxR1 variants.** *In vitro* activity assay of WT and acTrxR1 variants using a colorimetric **(A)** DTNB reduction assay measuring TNB production (412 nm) with 50 n*M* of each TrxR1 variant, **(B)** a 9,10-phenanthrenequinone reduction assay with 100 n*M* of each TrxR1 variant monitoring NADPH depletion (340 nm), or **(C)** a Trx1-insulin linked assay using 200 n*M* of TrxR1 and 6 μ*M* Trx1 monitoring NADPH depletion (340 nm). In the Trx1 insulin linked assay, TrxR1 reduces Trx1, which then reduces insulin before being reduced again by TrxR1. **(D)** The relative activity of the TrxR1 variants for each substrate, where the velocity (TNB/s or NADPH consumed/s) of WT TrxR1 is set to 1. These velocities were calculated from the data shown **(A**–**C)**. (*p < 0.05, **p < 0.01, ***p < 0.005). Error bars represent one standard deviation based on triplicate measurements. A no enzyme control has been subtracted from all enzyme assays shown. DTNB, 5,5′-dithiobis-(2-nitrobenzoic acid); NADPH, β-nicotinamide adenine dinucleotide 2′-phosphate; Trx1, thioredoxin 1; WT, wild type. To see this illustration in color, the reader is referred to the web version of this article at www.liebertpub.com/ars

**FIG. 4.**
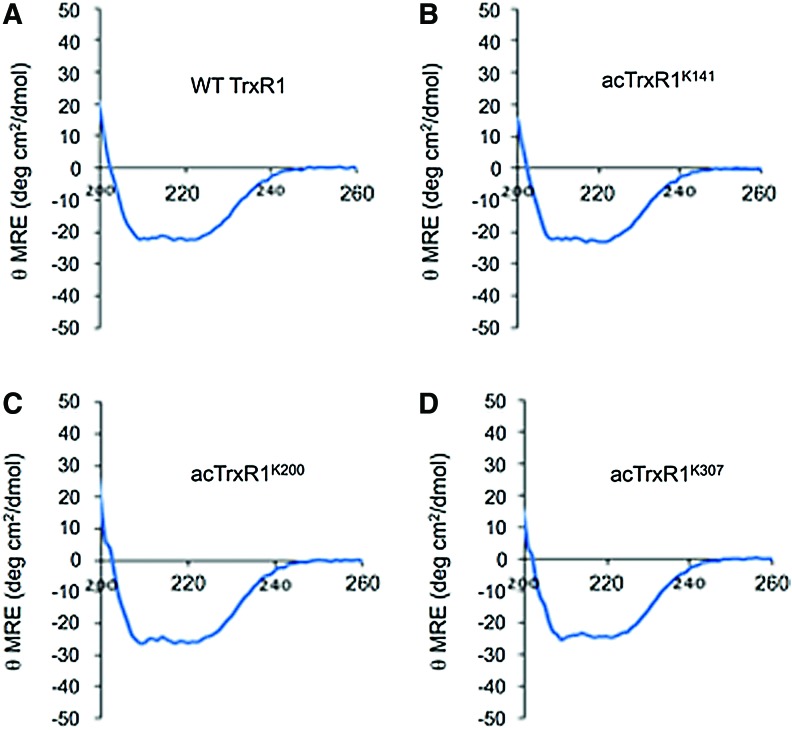
**CD spectra of TrxR1 variants.** CD spectra demonstrates properly folded WT TrxR1 **(A)** and acTrxR1 **(B–D)** variants, indicating no changes in protein folding caused by cotranslational incorporation of acK. Data are collected in units of mean residue ellipticity (in deg cm^2^/dmol). CD, circular dichroism. To see this illustration in color, the reader is referred to the web version of this article at www.liebertpub.com/ars

In comparison with WT TrxR1, each acTrxR1 variant had a statistically significantly higher activity on all substrates tested (Fig. 3). acTrxR1^K307^ was most active in DTNB assays, while acTrxR1^K200^ showed the highest activity with the quinone substrate (Fig. 3D), suggesting that acetylation may affect TrxR1 substrate preference. Although TrxR1 lacking the Sec residue is still active with DTNB at a low level, 9,10-phenanthrequinone reduction by TrxR1 is more Sec dependent (54). We observed that a disabled TrxR1 variant (Sec550Tyr) shows basal activity in the quinone assay (Fig. 3B). In agreement with the MS data, the activity data demonstrate that WT and acTrxR1s all contain Sec (Fig. 3B). Cotranslational incorporation of acK had no apparent effect on the proper folding of the enzyme (Fig. 4).

**FIG. 5.**
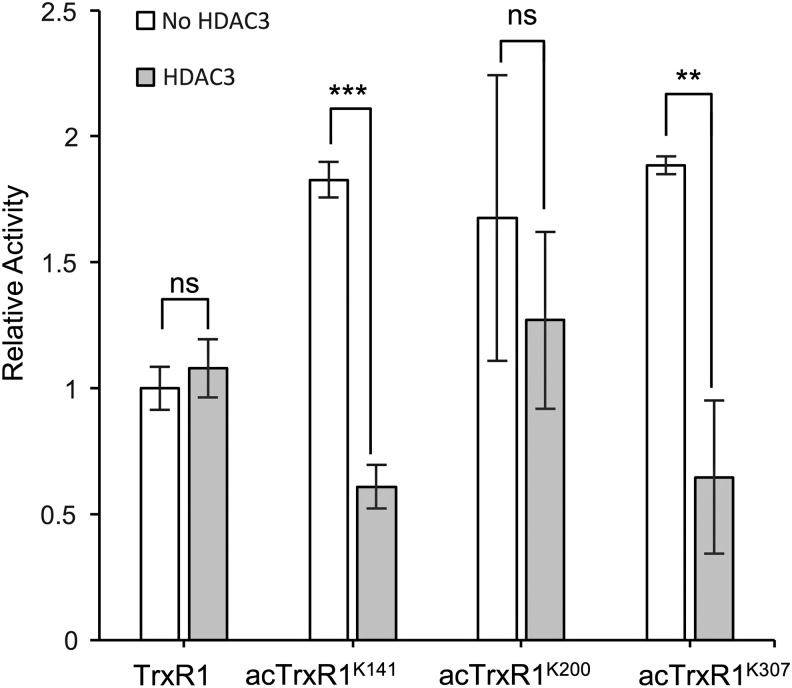
**Effect of HDAC on TrxR1 variants.** The *in vitro* activity of TrxR1 variants, using 3.75 n*M* TrxR1 incubated with or without HDAC3, was measured by monitoring TNB production (412 nm). The relative activity for each variant incubated with (*filled bars*) or without (*open bars*) HDAC3, where the initial velocity of unacetylated TrxR1 incubated without HDAC3 is set to 1. Error bars represent one standard deviation (**p < 0.01, ***p < 0.005, ns, not significant). All error bars represent one standard deviation of triplicate measurements. HDAC3, histone deacetylase 3.

We performed a series of TrxR1 activity assays with or without preincubation with histone deacetylase 3 (HDAC3). HDAC3 decreased the activity of the acTrxR1^K141^ and acTrxR1^K307^ variants to WT TrxR1 levels, but HDAC3 did not alter WT TrxR1 activity (Fig. 5). HDAC3 caused a slight decrease in the activity of acTrxR1^K200^, but this decrease was at the borderline of statistical significance (Fig. 5). Although not evident in the MS data, these data suggest that acTrxR1^K200^ may be either partially deacetylated or perhaps less accessible to HDAC3. The data show conclusively that acetylation is a reversible mechanism to enhance TrxR1 activity at sites K141 and K307. A single acetylation at K307 leads to a 2.7-fold increased TrxR1 activity (Fig. 3; Table 1).

### Acetylation alters TrxR1 quaternary structure

**FIG. 6.**
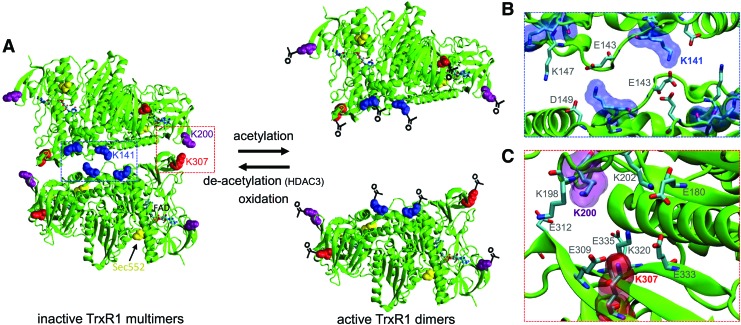
**Mechanistic basis for acetylation-dependent enhanced TrxR1 activity. (A)** Acetylation of K141 (*blue*), K200 (*purple*), or K307 (*red*) on the surface of TrxR1 dimers interferes with salt bridges and hydrogen bonds **(B**, **C)** in the TrxR1 tetramer interface. Close-up view of the interactions at the tetramer interface with **(B)** K141, **(C)** K200, and K307 [PBD: 4KPR (39) and 3EAN (5)]. To see this illustration in color, the reader is referred to the web version of this article at www.liebertpub.com/ars

In solution, catalytically active dimeric TrxR1 exists in equilibrium with inactive tetramers and higher order multimers (54). A structure of the TrxR1 tetramer (54) revealed that all three lysine acetylation sites are localized at the dimer–dimer interface [Fig. 6, PBD 4KPR (54) and 3EAN (6)]. In fact, acetylation will interfere with charge balanced hydrogen bond networks of glutamate or aspartate and lysine residues lining the tetramer interface (Fig. 6B, C). Acetylation at K141 disrupts a symmetrical hydrogen bond network at the core of the dimer–dimer interface between K141 and E143 (Fig. 6B). Additionally, acetylation at K200 or K307 likely perturbs a distinct and extensive hydrogen bond and salt bridge network between glutamate and lysine residues at the edges of the tetramer (Fig. 6C). In fact, a recent report identified acetylation at K147, which is also located in the TrxR1 dimer–dimer interface, in human lung cancer cells treated with the HDAC inhibitor suberoylanilide hydroxamic acid (52).

**FIG. 7.**
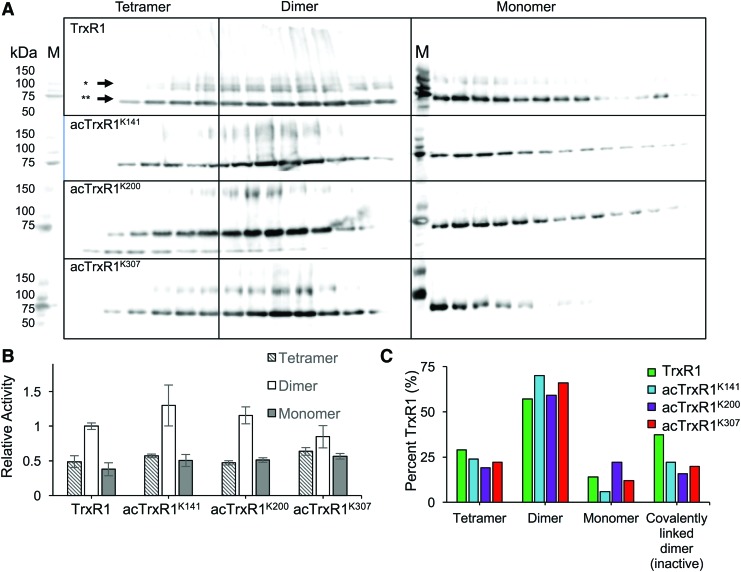
**Characterization of WT TrxR1 and acTrxR1 oligomerization. (A)** Different oligomeric states of the TrxR1 variants were separated in native conditions on a size exclusion column. His_6_-TrxR1 present in the fractions was visualized by Western blotting (anti-His). *Arrows* indicate TrxR1 subunits (**) or covalently linked and nonproductive TrxR1 dimers (*) formed during tetramerization. M indicates a molecular weight marker. **(B)** DTNB activity assays (100 n*M* TrxR1) demonstrated that high-activity dimers were successfully separated from low-activity tetramers and monomers. Relative activity is based on initial velocity (TNB/s), where the unacetylated dimer is set to 1. Error bars represent one standard deviation based on triplicate experiments. **(C)** The percent of TrxR1 existing as dimers, tetramers, monomers, or covalently linked inactive dimers was plotted for each TrxR1 variant. Percentages are calculated based on densitometry of the Western blot **(A)**. To see this illustration in color, the reader is referred to the web version of this article at www.liebertpub.com/ars

Catalytically active TrxR1 dimers were separated from low-activity monomers and tetramers for each TrxR1 variant by native size exclusion chromatography (Fig. 7). DTNB activity assays demonstrated the TrxR1 variants were successfully separated. In agreement with a previous study on the role of Trp114 oxidation in driving TrxR1 tetramer formation (54), the highest activity fractions contained dimers, while fractions containing monomers or tetramers showed markedly reduced activity (Fig. 7B). The oxidation of Trp114 also leads to a covalent linkage between two TrxR1 monomers (54), forming a nonproductive and covalently linked dimer. As evident in the Western blot (Fig. 7), we observed fewer covalently linked inactive TrxR1 subunits in the acetylated TrxR1 variants compared with WT TrxR1 (Fig. 7A, C). Western blot analysis of the fractions confirms that a larger fraction of the acTrxR1s eluted as catalytically active dimers compared with WT TrxR1 (Fig. 7C, Supplementary Table S2). The data further suggest that the acTrxR1 and WT TrxR1 dimer fractions have similar activity (Fig. 7B), and the increased activity observed in the bulk measurements (Fig. 3) is attributable to the increased population of active dimers in acTrxR1, decreased tetramer formation in acTrxR1 (Fig. 7C, Supplementary Table S2), and decreased amounts of nonproductive covalently linked dimers in acTrxR1 compared with WT TrxR1 (Fig. 7C). The data suggest a novel mechanism (Fig. 6), through which acetylation reduces tetramer/multimer formation and promotes the formation of active TrxR1 dimers.

## Discussion

### Acetylation controls TrxR1 structure and activity

Lysine acetylation is found in all domains of life (10, 40, 51) with 36,000 acetylation sites identified in human, rat, and mouse cells (7, 21). The human acetylome is enriched in proteins that form macromolecular complexes (7). Acetylation of p53 blocks its oligomerization leading to nuclear export (23). Histone acetylation prevents oligomerization, inhibiting chromatin condensation and increasing transcriptional activity (43). Although not previously known for thioredoxin reductase, our data indicate that a similar mechanism may allow cells to control the pool of active molecules by suppressing TrxR1 oligomerization (Fig. 6). Acetylation of TrxR1 may also counteract oxidative inactivation of the enzyme. Oxidative modification at Trp114 (54), centrally located in the dimer–dimer interface, was shown to reduce TrxR1 activity by promoting TrxR1 oligomerization, which represents a counteracting mechanism relative to acetylation (Fig. 6) (54). Indeed, the data suggest that TrxR1 acetylation may enable reactivation of oxidatively damaged TrxR1 multimers (Fig. 7). In addition, acetylation of the TrxR1 substrate, Trx1, increases the activity of Trx1 through a currently unknown mechanism (46). Acetylation of another protein target of the Trx system, peroxiredoxin (Prx), prevents superoxidation of the Prx protein, increases its reduction activity, and prevents the formation of high-molecular-weight complexes with Prx and other proteins (33).

### Relevance to human disease

The ability to regulate TrxR1 activity *via* acetylation will have relevance to major human diseases, including neurodegeneration, cancer, diabetes, and aging (37), which are characterized or complicated by elevated ROS. Decreased TrxR1 activity without changes in TrxR1 levels has been implicated in age-related macular degeneration and glaucoma resulting from overexpression of amyloid β peptides (25), indicating that post-translational modifications control TrxR1 activity. Differential acetylation of TrxR1 was also documented in human cells (7) and in a mouse model of cardiomyopathy (2). Initial findings have implicated TrxR1 regulation by deacetylation in diabetes. In hyperglycemic rats, physical association with HDAC6 and decreased acetylation of TrxR1 correlated with reduced TrxR1 activity (26) in agreement with our data.

In several forms of cancer, TrxR1 expression and activity are used as diagnostic markers for early detection (8, 39). As our data indicate, acetylation increases TrxR1 activity, and future work will include investigating TrxR1 acetylation as a potential diagnostic marker. Cells can overcome ROS generating cancer therapies and turn off apoptotic pathways by overexpressing TrxR1 (36). In addition, inhibiting the Trx system has been shown to increase the effectiveness of radiation therapy in treating some forms of breast cancer (34). Oxidative stress is known to reduce TrxR1 activity by inducing tetramer formation (54). Previous data suggest that the cell responds to oxidative stress by acetylating TrxR1, perhaps enabling resistance to therapies that generate ROS (2). We hypothesize that, in the cell, acetylation of TrxR1 reduces the oxidative stress-induced tetramerization of TrxR1 (54) generated by radiation and chemotherapies (15). Together, these studies suggest that identifying and inhibiting TrxR acetylases may serve as another route to increase the effectiveness of cancer therapies.

### Impacts of enhanced TrxR activity on cellular signaling

The Trx system is directly involved in cellular signaling linked to cell proliferation and viability (27). A classic example includes the role of reduced Trx1 in inhibiting apoptosis. Reduced Trx1 inhibits apoptosis signal-regulating kinase (apoptosis signal-regulating kinase 1 [ASK1]) by directly binding to the kinase (36). Inhibition of TrxR with 1-chloro-2,4-dinitrobenzene in HEK 293 cells activated ASK1 leading to apoptosis (36). This provides a direct link between TrxR activity and its negative regulation of apoptosis. In addition, Trx1 activity is involved in activating transcription factors involved in cell proliferation (27), such as nuclear factor-κB (20) and activator protein 1 (19). We have definitively shown that acetylation increases the intrinsic activity of TrxR1 (Fig. 3A, B), which leads to an increased rate of Trx1 reduction, thus increasing the activity of the Trx system in reduction of cellular targets, such as insulin (Fig. 3C). Together, these observations suggest that acetylation signaling on TrxR1 may be involved in inhibiting apoptosis and regulating cell viability by increasing the overall activity of the Trx system.

### Codon recoding and codon reassignment mechanisms are mutually orthogonal

Efficient protein production with more than 20 amino acids is a major challenge in the area of genetic code expansion. Protein production at 1.5 mg/L culture with two different ncAAs in the cell was recently demonstrated by reassigning a UAG codon and a quadruplet codon (AGUA) in the same gene (48). This experiment relied on coexpression of a mutant orthogonal ribosome alongside the endogenous ribosome. Translation products without one or both of the two ncAAs were also observed (48), and it is unclear why this mutant ribosome would be immune to frame-shifting at the site of the four-base codon, which is well documented (13, 32).

In an independent experiment, a GFP reporter containing two different ncAAs was produced at 1–10 mg/L culture by reassigning both UAG and UAA stop codons (47). The corresponding “wild-type” GFP variant was produced at 56 mg/L culture (22). These data suggest that GFP with 22 different amino acids was produced at 6- to 50-fold lower levels compared with a GFP containing only the 20 natural amino acids. In contrast, here we established protein synthesis with 22 different amino acids in a recombinant human protein for the first time at >2 mg/L culture. Our data indicate we achieved equal efficiency to standard protein production with 20 amino acids.

A key bottleneck in genetic code expansion is a limited availability of “reassignable” codons (31). Traditional genetic code expansion systems alter the meaning of the UAG codon at each instance of that codon in the entire transcriptome (1, 18). The Sec insertion machinery recodes the UGA codon only at genetic loci that are specified by a downstream selenocysteine insertion sequence (SecIS) element (17) (Fig. 1). This means that UGA normally stops protein synthesis, but at specific sites determined by the position of SecIS, the Sec machinery is recruited to change the meaning of UGA from stop to Sec. Sec is not hardwired to UGA (3, 29), and we have previously shown that by simply mutating the tRNA^Sec^ anticodon, each stop codon and 15 sense codons can be recoded with high fidelity to Sec (3). A recent study showed that recoding UAG to Sec using an Sec-tRNA^Sec^ with the corresponding UAG-decoding anticodon results in increased UAG translation with Sec in an RF1-deficient *E. coli* strain (5). We have shown here (Table 1) and previously (14) that despite enhanced UAG read-through per cell in the same RF1-deficient *E. coli* strain (Supplementary Fig. S2), we are able to produce ncAA-containing proteins more efficiently in BL21 as evidenced by 5- to 100-fold higher protein yields per liter *E. coli* culture. This is likely due to the decreased fitness of the RF1-deficient *E. coli* strain, resulting in slower growth (24), and less overall protein production. Together, these data suggest that with an efficient genetic code expansion system, RF1 competition (and translational stopping at UAG) is offset by higher cell densities and overall more efficient protein production in BL21 compared to RF1-deficient *E. coli.*

Because the Sec system shows surprising efficiency at stop codon (5) and sense codon recoding (3), we are currently engineering this system to incorporate other ncAAs (41). The data here are vital to this engineering effort as they demonstrate that the Sec codon recoding machinery is compatible with, and orthogonal to, traditional genetic code expansion systems based on UAG codon reassignment. In addition, because our system for protein synthesis with 22 amino acids uses normal three-base codons and the native ribosome, it is highly efficient and will be portable to other host expression systems, including mammalian cells (11, 44) with minimal intervention.

## Materials and Methods

### Bacterial strains and plasmids

Site-specific insertion of acK into proteins relies on translational read-through of the stop codon UAG (Fig. 1). In an attempt to enhance UAG translation with acK, we expressed TrxR1 variants in an *E. coli* RF1 deletion strain and in *E. coli* BL21 (DE3) (C2527H; New England Biolabs, Ipswich, MA). We used an *E. coli* ΔRF1 strain [C321.ΔA.exp, from G. Church (24) *via* Addgene strain #49018] that also has all genomic TAG codons mutated to TAA (24). pET-pylT-GFP, containing sfGFP with an in frame UAG codon at position 2, and pylT as described previously (16), was used for accessing acK incorporation in *E. coli* ΔRF1 (Supplementary Fig. S2).

Site-specific insertion of Sec relies on the translational read-through and site-specific recoding of the stop codon UGA (Fig. 1). *E. coli'*s native Sec insertion machinery was used to site specifically insert Sec as previously (3). *E. coli* naturally produces Sec-tRNA^Sec^_UCA_ that binds to a specialized elongation factor (SelB), which in turn recognizes an RNA-hairpin loop (SecIS) downstream of the recoded UGA codon (Fig. 1). Human TrxR1 was recombinantly expressed in *E. coli* by placing an *E. coli* SecIS sequence (derived from the *E. coli fdhF* gene) in the 3′ untranslated region (3′ UTR) of the TrxR1 expression construct, as previously (3).

To develop a system capable of cotranslational incorporation of both Sec and acK in the same polypeptide, human TrxR1 (isoform 4), including the 3′ *fdhF* SecIS and an in-frame UGA codon (Sec550), was PCR amplified from pRSF-TrxR1-SerS (3) with primers (TRXF-*Nde*I 5′-CATATGTCCTGCGAAGACGGTCGTGCGC-3′, TRXR-SacI 5′-GAGCTCTCGGCCGCATAGGCTAACGATTG-3′). The PCR product was digested with *Nde*I/*Sac*I (NEB) and ligated to pET-pylT (16) digested with *Nde*I/*Sac*I to give pET-pylT-TrxR1 (WT). Quickchange PCR, as described previously (9), was conducted on pET-pylT-TrxR1 (WT) to introduce UAG codons in the TrxR1 expression construct at positions 141, 200, or 307 (residue numbers are based on isoform four numbering). pKTS-acKRS1 (45) containing an engineered *N_ɛ_*-acetyl-lysine tRNA synthetase (16), or pTech-acKRS-tRNA^Pyl-opt^ containing acKRS and tRNA^Pyl-opt^ (12), was also required for site-specific acK incorporation. The tRNA^Pyl-opt^ mutant (12) was shown to increase acK incorporation 3-fold relative to the earlier established systems (30, 45). pRSF-TrxR1-SerS (3) containing UAC at codon 550 was used for producing inactive TrxR1 Sec550Tyr.

### TrxR1 production and purification

The human TrxR1 variants were overexpressed from the plasmids pET-pylT-TrxR1 in *E. coli* ΔRF1 (24) or *E. coli* BL21 DE3 that had been cotransformed with pKTS-acKRS1 (45) or pTech-acKRS-tRNA^Pyl-opt^ (12). Starting from a single colony after transformation on selective agar plates, cells were grown at 37°C overnight in a 25 mL preculture containing lysogeny broth (LB) selective medium (ampicillin 100 μg/mL, kanamycin 25 μg/mL for pKTS-acKRS1; or ampicillin 100 μg/mL, chloramphenicol 34 μg/mL for pTech-acKRS-tRNA^Pyl-opt^). Cells were grown at 37°C in 1 L LB selective medium (ampicillin 100 μg/mL, kanamycin 25 μg/mL for pKTS-acKRS1; or ampicillin 100 μg/mL, chloramphenicol 34 μg/mL for pTech-acKRS-tRNA^Pyl-opt^) supplemented with 10 μ*M* sodium selenite (214485; Sigma) and 2.5 m*M N_ɛ_*-acetyl-lysine (A2010; Sigma) following inoculation with a 25 mL preculture. An additional 1.5 m*M* acK was added at OD_600_ = 1.0, for a total of 4 m*M N_ɛ_*-acetyl-lysine. Because optimal selenoprotein expression requires late induction (3), at OD_600_ = 1.2, the temperature was shifted to 20°C and protein expression was then induced at OD_600_ = 1.5 with 100 μ*M* or 1 m*M* isopropyl β-d-1-thiogalactopyranoside (I6758; Sigma) and continued for 18 h shaking at 20°C.

After the cells were harvested by centrifugation, the cell pellet was resuspended in 30 mL phosphate buffer (100 m*M* potassium phosphate, pH 7.2, 10% glycerol). The cells were supplemented with lysozyme (0.05 mg/mL, LDB0308; Biobasic) and subsequently disrupted by sonication (Q125; QSonica). The centrifuged lysate (6250 *g*, 1 h, 4°C) was purified by affinity chromatography using 1 mL Ni-NTA resin (30230; Qiagen) in a gravity flow column equilibrated with 25 mL phosphate buffer. After washing with 50 mL phosphate buffer supplemented with 45 m*M* imidazole, the proteins were eluted with 5 × 1 mL phosphate buffer (230 m*M* imidazole). Protein concentration was assessed by the Bradford protein assay (#5000006; Bio-Rad) at 595 nm according to the manufacturer's instructions.

Following affinity chromatography, the TrxR1 variants were further purified by size-exclusion chromatography. TrxR1 variants were purified in an AKTA Pure L1 fast protein liquid chromatography (FPLC) system (29018225; GE Healthcare) using a Superdex^™^ 200 Increase 10/300 GL column (28990944; GE Healthcare) pre-equilibrated in 20 m*M* sodium phosphate, 150 m*M* sodium chloride, pH 7.5, with a flow rate of 0.3 mL/min. Elutions were collected in 0.5 mL fractions.

### *In vitro* TrxR1 activity assay: DTNB

Enzymatic activity of purified TrxR1 variants was assessed colorimetrically by monitoring NADPH (N7505; Sigma**)-**dependent reduction of Ellman's reagent (DTNB, D8130; Sigma). A plate reader (Synergy H1 Hybrid Multi-Mode Reader, 11-120-534; BioTek) autodispenser was used to add DTNB (in phosphate buffer) to each well to start the reactions, which included final concentrations of 5 m*M* DTNB, 300 μ*M* NADPH, and 50 n*M* (Fig. 3A) or 100 n*M* (Fig. 7B) TrxR1 in a final volume of 100 μL per well. This reaction was monitored at 412 nm every minute for 50 min. Reactions were performed in triplicate. For all activity assays, error bars display one standard deviation based on at least triplicate experiments. A control lacking the TrxR1 enzyme was conducted in triplicate and has been subtracted from all enzyme assays.

### *In vitro* TrxR1 activity assay: 9,10-phenathrenequinone

Enzymatic activity of purified TrxR1 variants was assessed colorimetrically by following oxidation of NADPH resulting from TrxR1-mediated and Sec-dependent reduction of 9,10-phenathrenequinone (275034; Aldrich). NADPH consumption was monitored by incubating 100 n*M* TrxR1 with 300 μ*M* NADPH and 50 μ*M* 9,10-phenanthrenequinone in phosphate buffer (100 m*M* potassium phosphate, pH 7.0, 1 m*M* ethylenediaminetetraacetic acid [EDTA, E9884; Sigma]). TrxR1 and NADPH in phosphate buffer with EDTA were added to the wells of a 96-well plate. A 96-well plate reader autodispenser was used to add 9,10-phenanthrenequinone in phosphate buffer to each well to start the reaction with a final volume of 100 μL. The assay mixture was monitored at 340 nm every minute for 2 h, in a 96-well plate reader. A no enzyme control was conducted in triplicate and subtracted from the TrxR1 reactions. Reactions were performed in triplicate.

### HDAC3 assays

WT and acTrxR1 variants (0.75 μ*M*) were incubated with 4 μ*M* HDAC3 (SRP2072; Sigma) for 2 h at 37°C. Following this incubation, the enzymatic activity of TrxR1 variants incubated with or without HDAC3 was assessed colorimetrically by the *in vitro* DTNB activity assay described above, with a final TrxR1 concentration of 3.75 n*M*. Reactions were performed in triplicate, and error bars represent one standard deviation.

### Insulin linked TrxR1 activity assays

The enzymatic activity of TrxR1 on recombinant human Trx1 (T8690; Sigma) was assessed by following the oxidation of NAPDH (340 nm) resulting from TrxR1-mediated reduction of Trx1. In this insulin linked assay, Trx1 then reduces recombinant human insulin (91077C; Sigma) before being reduced by TrxR1 again. TrxR1 (200 n*M*) was incubated with 50 μ*M* NADPH, 1 m*M* EDTA, 80 μ*M* insulin, and 6 μ*M* Trx1 in phosphate buffer in a volume of 200 μL in wells of a 96-well plate. The reaction was started by the addition of Trx1 and insulin. The reactions were monitored at 340 nm every minute for 10 min using a plate reader. Reactions were performed in triplicate. Error bars represent one standard deviation. A no TrxR1 control (performed in triplicate) has been subtracted from all reactions.

### Statistical analysis

All errors bars represent one standard deviation. All *p*-values were derived from an analysis of variance one-way statistical analysis of data produced in triplicate. Initial velocity calculations in Table 1 are calculated from the linear phase of the activity curves (Fig. 3).

### Mass spectrometry

Mass spectrometric analyses of purified TrxR1 variants were performed at the MALDI mass spectrometry facility (The University of Western Ontario) for MALDI-MS and the UWO Biological Mass Spectrometry Laboratory (The University of Western Ontario) or the W.M. Keck Biotechnology Resource Laboratory (Yale University) for LC-MS/MS. TrxR1 variants were run on a 15% sodium dodecyl sulfate (SDS) gel, Coomassie stained, and prepared for MALDI-MS or LC-MS/MS by digestion with trypsin protease. A MassPREP automated digester station (PerkinElmer) was used to digest the TrxR1 variants with 5 ng/μL trypsin (Promega). Coomassie-stained gel pieces were destained with 50 m*M* ammonium bicarbonate and 50% acetonitrile. TrxR1 variants were then reduced with 10 m*M* dithiothreitol, alkylated with 55 m*M* acrylamide, and digested with trypsin. LC-MS/MS was performed using a Q-Tof Micro mass spectrometer (Waters) equipped with a Z-spray source and run in positive ion mode (+0.1% formic acid) or using Thermo Scientific LTQ-Orbitrap XL mass spectrometer. MALDI-MS was performed using an AB Sciex 5800 TOF/TOF system, MALDI TOF-TOF (Framingham, MA) equipped with a 349 nm Nd:YLF OptiBeam On-Axis laser using a laser pulse rate of 400 Hz and reflectron positive mode. The MALDI matrix used was α-cyano-4-hydroxycinnamic acid, prepared as 5 mg/mL in 6 m*M* ammonium phosphate monobasic, 50% acetonitrile, 0.1% trifluoroacetic acid, and was mixed with the samples at a 1:1 ratio (v/v). ICP-MS was performed to quantify the amount of selenium per amount of protein for TrxR1 variants at Biotron Analytical Services (The University of Western Ontario).

### Western blot analysis of TrxR1 expression

Purified TrxR1 proteins were resuspended in 1 × SDS loading buffer (250 m*M* Tris/HCl pH 6.8, 40% glycerol [v/v], 10% SDS [w/v], 0.05% bromophenol blue [w/v], 5% 2-mercaptoethanol) and loaded in 10% SDS-polyacrylamide gel electrophoresis. The blot was carried out with a TransBlot Turbo Transfer System (BioRad). Anti-His antibodies (from Mouse, H1029; Sigma) and mouse immunoglobulin G horseradish peroxidase linked whole antibody (from sheep, GENA931; GE Healthcare) were used. Chemiluminescent signal detection was performed on a ChemiDoc MP system (BioRad).

### Size exclusion chromatography

TrxR1 variants and proteins from a high-molecular-weight calibration kit (28403842; GE Healthcare) were individually separated in an AKTA Pure L1 FPLC system using a Superdex 200 Increase 10/300 GL column pre-equilibrated in 20 m*M* sodium phosphate, 150 m*M* sodium chloride, pH 7.5. Elutions were collected in 0.5 mL fractions. The high-molecular-weight calibration kit used the following proteins: ovalbumin (43 kDa, 3 mg/mL), conalbumin (75 kDa, 3 mg/mL), aldolase (158 kDa, 3 mg/mL), ferritin (440 kDa, 1 mg/mL), thyroglobulin (669 kDa, 3 mg/mL), and blue dextran (2000 kDa, 1 mg/mL).

## Supplementary Material

Supplemental data

Supplemental data

Supplemental data

Supplemental data

Supplemental data

Supplemental data

Supplemental data

Supplemental data

## Abbreviation Used

3′ UTR: 3′ untranslated region
acK: *N_ɛ_*-acetyl-lysine
acKRS: mutant pyrrolysyl-tRNA synthetase (*N_ɛ_*-acetyl-lysine-tRNA synthetase)
acTrxR1: acetylated thioredoxin reductase 1
ASK1: apoptosis signal-regulating kinase 1
DTNB: 5,5′-dithiobis-(2-nitrobenzoic acid)
EDTA: ethylenediaminetetraacetic acid
FPLC: fast protein liquid chromatography
HDAC3: histone deacetylase 3
ICP-MS: inductively coupled plasma mass spectrometry
LB: lysogeny broth
LC-MS/MS: liquid chromatography tandem mass spectrometry
MALDI-MS: matrix-assisted laser desorption/ionization mass spectrometry
MS: mass spectrometry
NADPH: β-nicotinamide adenine dinucleotide 2′-phosphate
ncAAs: noncanonical amino acids
Prx: peroxiredoxin
RF1: release factor 1
ROS: reactive oxygen species
SDS: sodium dodecyl sulfate
Sec: selenocysteine
SecIS: selenocysteine insertion sequence
Trx1: thioredoxin 1
TrxR1: thioredoxin reductase 1
WT: wild type
