# Supplementary material for: Acetylation Regulates Thioredoxin Reductase Oligomerization and Activity
Source: Antioxid Redox Signal. 2018 Aug 1;29(4):377–88. doi: 10.1089/ars.2017.7082 (PMC6025699; doi:10.1089/ars.2017.7082)
Supplement: Supplemental data [file Supp_Fig1.pdf]

Supplementary Data

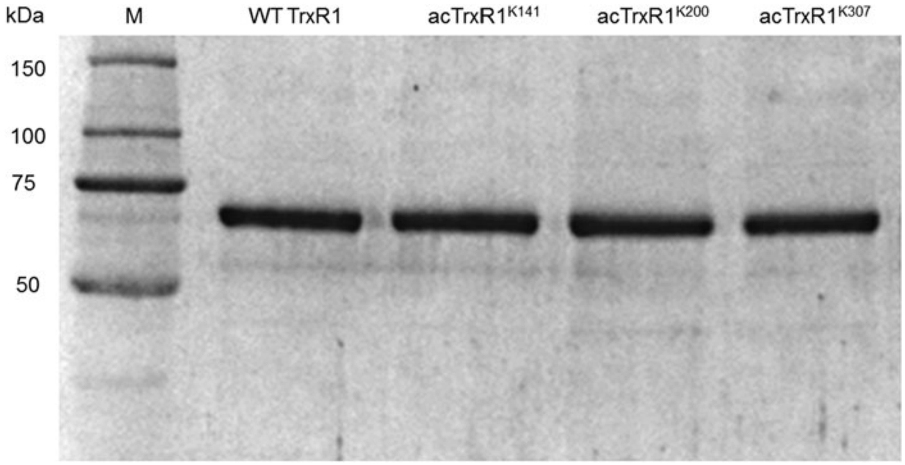

SUPPLEMENTARY FIG. S1. Purified WT and acTrxR1 variants. His-tagged WT TrxR1 and acetylated TrxR1 (acTrxR1) variants were visualized on a Coomassie-stained sodium dodecyl sulfate gel showing successful purification of full-length (62 kDa) TrxR1 variants (isoform 4). The variants are indicated above each lane, while M represents a molecular weight marker. TrxR1, thioredoxin reductase 1; WT, wild type.
