# Supplementary material for: Acetylation Regulates Thioredoxin Reductase Oligomerization and Activity
Source: Antioxid Redox Signal. 2018 Aug 1;29(4):377–88. doi: 10.1089/ars.2017.7082 (PMC6025699; doi:10.1089/ars.2017.7082)
Supplement: Supplemental data [file Supp_Fig2.pdf]

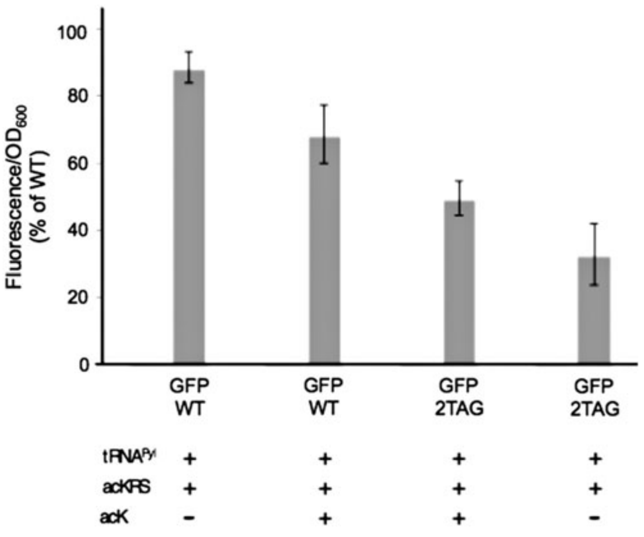

SUPPLEMENTARY FIG. S2. GFP reporter detecting UAG read-through. Protein expression levels for GFP reporters in *Escherichia coli* ΔRF1. GFP fluorescence levels were measured in an *E. coli* ΔRF1 background strain expressing acKRS and tRNA^{Pyl}. Expression of WT GFP and GFP with a UAG codon at position 2 (2TAG) was compared in the presence or absence of acK. acK, N_{ε}-acetyl-lysine; acKRS, mutant pyrrolysyl-tRNA synthetase (N_{ε}-acetyl-lysine-tRNA synthetase); RF1, release factor 1.
