# Supplementary material for: Acetylation Regulates Thioredoxin Reductase Oligomerization and Activity
Source: Antioxid Redox Signal. 2018 Aug 1;29(4):377–88. doi: 10.1089/ars.2017.7082 (PMC6025699; doi:10.1089/ars.2017.7082)
Supplement: Supplemental data [file Supp_Fig3.pdf]

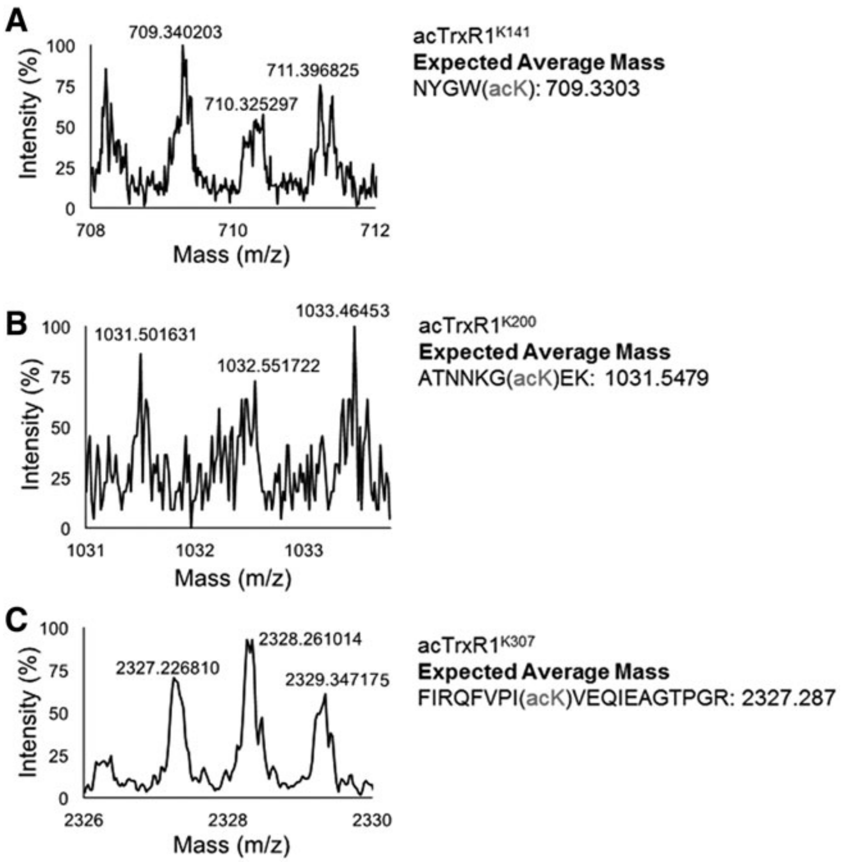

SUPPLEMENTARY FIG. S3. MALDI-MS analysis confirms acetylation of acTrxR1 variants. Peaks for tryptic peptides corresponding to acetylated lysine at position 141 **(A)**, 200 **(B)**, and 307 **(C)** were observed. The expected average mass reported refers to the monoisotropic mass (all carbons ^{12}C). Peptides containing 1 or 2 ^{13}C isotope(s) are also shown, and should have the expected average mass +1 or +2, respectively. MALDI-MS, matrix-assisted laser desorption/ionization mass spectrometry.
