# Supplementary material for: Acetylation Regulates Thioredoxin Reductase Oligomerization and Activity
Source: Antioxid Redox Signal. 2018 Aug 1;29(4):377–88. doi: 10.1089/ars.2017.7082 (PMC6025699; doi:10.1089/ars.2017.7082)
Supplement: Supplemental data [file Supp_Fig6.pdf]

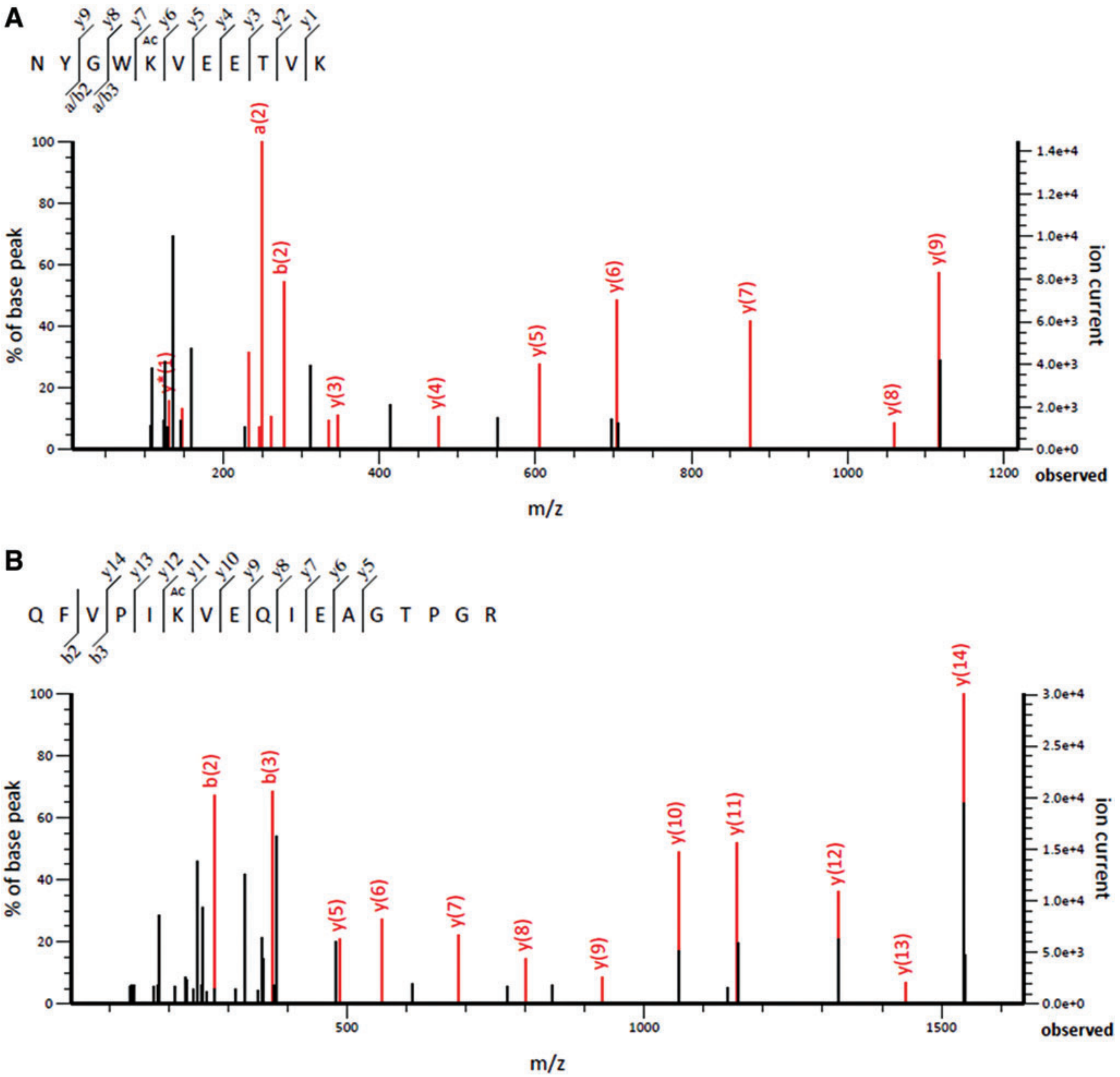

SUPPLEMENTARY FIG. S6. LC-MS/MS confirming genetically encoded acK incorporation into acTrxR1^{K141} and acTrxR1^{K307}. Trypsin digested WT and acTrxR1 variants were analyzed by LC-MS/MS. A peptide from trypsin digested TrxR1 demonstrating acK incorporation for acTrxR1^{K141} (**A**) and acTrxR1^{K307} (**B**). Acetylated lysine is indicated by K with AC superscript.
