# Supplementary material for: Acetylation Regulates Thioredoxin Reductase Oligomerization and Activity
Source: Antioxid Redox Signal. 2018 Aug 1;29(4):377–88. doi: 10.1089/ars.2017.7082 (PMC6025699; doi:10.1089/ars.2017.7082)
Supplement: Supplemental data [file Supp_Fig4.pdf]

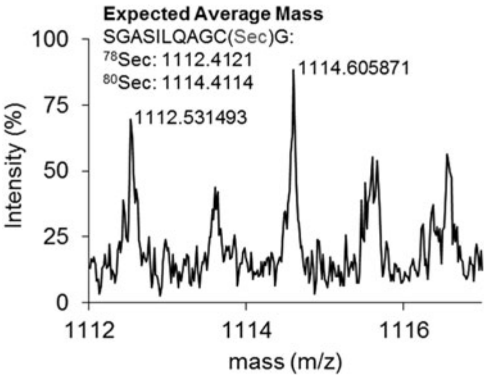

SUPPLEMENTARY FIG. S4. Confirmation of Sec incorporation in WT TrxR1 by MALDI-MS analysis of the tryptic digested protein. Peaks for peptides containing two Se isotopes (^{78}Se, ^{80}Se) were observed. Sec, selenocysteine.
