# Supplementary material for: Acetylation Regulates Thioredoxin Reductase Oligomerization and Activity
Source: Antioxid Redox Signal. 2018 Aug 1;29(4):377–88. doi: 10.1089/ars.2017.7082 (PMC6025699; doi:10.1089/ars.2017.7082)
Supplement: Supplemental data [file Supp_Table1.pdf]

Supplementary Table S1. Inductively Coupled Plasma Mass Spectrometry Selenium Content Determination for Thioredoxin Reductase 1 Preparations

TrxR1 variant	*Protein (μM)*	*Selenium (μM)*	*Sec occupancy (%)*[a]
TrxR1 WT	25.8	21.5 ± 2.4	83 ± 9
$acTrxR1^{K141}$	24.2	25.0	103
$acTrxR1^{K200}$	25.2	25.5 ± 2.3	101 ± 9
$acTrxR1^{K307}$	22.6	21.8 ± 2.2	96 ± 10

[a]The Sec occupancy is calculated as a ratio of the amount of Se detected by ICP-MS to the amount of protein in each sample. The data are based on triplicate measurements.

acTrxR1, acetylated TrxR1; ICP-MS, inductively coupled plasma mass spectrometry; Sec, selenocysteine; TrxR1, thioredoxin reductase 1; WT, wild type.
