# Supplementary material for: Acetylation Regulates Thioredoxin Reductase Oligomerization and Activity
Source: Antioxid Redox Signal. 2018 Aug 1;29(4):377–88. doi: 10.1089/ars.2017.7082 (PMC6025699; doi:10.1089/ars.2017.7082)
Supplement: Supplemental data [file Supp_Fig5.pdf]

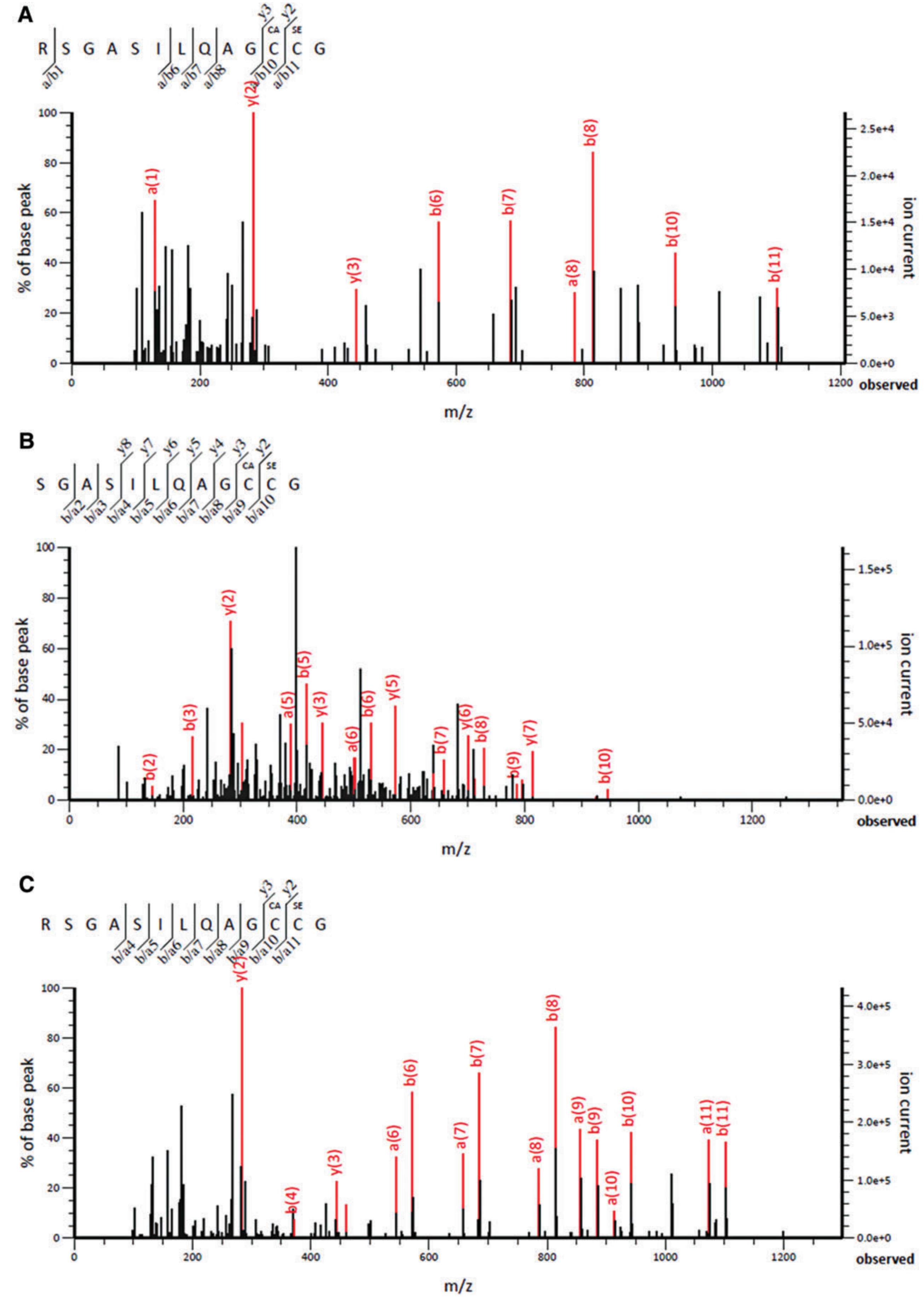

SUPPLEMENTARY FIG. S5. LC-MS/MS confirming Sec incorporation for WT TrxR1, acTrxR1^{K141}, and acTrxR1^{K307} variants. Trypsin digested WT and acTrxR1 variants were analyzed by LC-MS/MS. A peptide from trypsin digested TrxR1 demonstrating Sec incorporation for WT TrxR1 (**A**), acTrxR1^{K141} (**B**), and acTrxR1^{K307} (**C**). Sec is shown as C with Se superscript, while cysteine is noted as C with CA superscript. acTrxR1, acetylated TrxR1; LC-MS/MS, liquid chromatography tandem mass spectrometry; Sec, selenocysteine.
