# Supplementary material for: Acetylation Regulates Thioredoxin Reductase Oligomerization and Activity
Source: Antioxid Redox Signal. 2018 Aug 1;29(4):377–88. doi: 10.1089/ars.2017.7082 (PMC6025699; doi:10.1089/ars.2017.7082)
Supplement: Supplemental data [file Supp_Table2.pdf]

Supplementary Table S2. Percentage of Total Thioredoxin Reductase 1 Existing in High-Activity Dimers or Low-Activity Tetramers or Monomers

TrxR1 variants	*TrxR1 tetramer (% total TrxR1)*	*TrxR1 dimer (% total TrxR1)*	*TrxR1 monomer (% total TrxR1)*
WT	29	57	14
$acTrxR1^{K141}$	24	70	6
$acTrxR1^{K200}$	22	59	22
$acTrxR1^{K307}$	19	66	12

A Western blot was conducted on 0.5 mL elutions of TrxR1 variants from a size exclusion column (Fig. 7). DTNB activity assays were used to determine where catalytically active dimers and inactive tetramers and monomers eluted from the size exclusion column (Fig. 7). The percentage of TrxR1 existing as tetramers, dimers, and monomers was calculated based on densitometry of the Western blots (Fig. 7).

DTNB, 5,5′-dithiobis-(2-nitrobenzoic acid).
